# Identification of cell morphology parameters from automatic hematology analyzers to predict the peripheral blood CD34-positive cell count after mobilization

**DOI:** 10.1371/journal.pone.0174286

**Published:** 2017-03-20

**Authors:** Saeam Shin, Sung Ran Cho, Sinyoung Kim, Jong Rak Choi, Kyung-A Lee

**Affiliations:** 1 Department of Laboratory Medicine, Yonsei University College of Medicine, Seoul, Korea; 2 Department of Laboratory Medicine, Hallym University College of Medicine, Kangnam Sacred Heart Hospital, Seoul, Korea; 3 Department of Laboratory Medicine, Ajou University School of Medicine, Suwon, Korea; European Institute of Oncology, ITALY

## Abstract

Optimal timing of apheresis initiation is important for maximizing the hematopoietic stem cell (HSC) yield. This study aimed to identify useful parameters from automatic hematology analyzers for predicting the peripheral blood CD34+ cell count after mobilization. We prospectively enrolled 53 healthy donors and 72 patients, and evaluated 43 cell morphology parameters from Unicel DxH800 (Beckman Coulter, USA) and Advia 2120i (Siemens Healthcare Diagnostics, USA). The correlation of each parameter with the CD34+ cell count in pre-apheresis blood samples was analyzed. The delta neutrophil index (DNI) from Advia 2120i, standard deviation of volume of neutrophils and monocytes (SD-V-NE and SD-V-MO), standard deviation of conductivity of neutrophils and monocytes (SD-C-NE and SD-C-MO), mean conductivity of neutrophils and monocytes (MN-C-NE and MN-C-MO), and standard deviation of axial light loss of neutrophils and monocytes (SD-AL2-NE and SD-AL2-MO) from DxH800 showed significant correlations with the CD34+ cell count. SD-V-NE, SD-C-NE, and SD-C-MO showed good or fair area under the curve values for the prediction of the CD34+ cell count. SD-V-NE, SD-C-NE, and SD-C-MO from DxH800 will provide rapid, useful information for the initiation of apheresis after mobilization.

## Introduction

Hematopoietic stem cell (HSC) transplantation is widely used in the curative treatment of a number of malignancies and immunologic diseases [1]. For successful transplantation, an adequate amount of HSC infusion is necessary [2]. Poor mobilizers with failure to achieve the minimal amount of HSCs for engraftment show prolonged engraftment time and worse prognosis [3]. The timing of the initiation of the apheresis schedule is important for maximizing the HSC yield in an apheresis product [4]. The most accurate and recommended method to predict the HSC yield is measuring the CD34+ cell count in peripheral blood (PB); however, this requires an additional 1–2 hours of analysis time, a flow-cytometer and reagents, and experienced technicians [5]. Therefore, surrogate markers obtained by an easy and inexpensive method can aid in daily HSC monitoring and allow fast medical decision-making for apheresis initiation.

Automatic hematologic analyzers differentiate leukocyte subpopulations using the principles of electrical impedance, radiofrequency conductivity, light scattering, and/or cytochemistry [6]. In the process, variable cell morphology parameters, derived from multiple channels and reflecting the cell size, cytoplasmic granularity, and nuclear lobularity, are obtained [6]. Recently, hematopoietic progenitor cells (HPCs) measured by the Sysmex hematology analyzer (Sysmex, Kobe, Japan) have been used as an estimate of immature cells in PB and as a marker for initiation of apheresis [7,8]. The HPC count by the Sysmex analyzer is determined by the combination of cell size and density, along with a resistance characteristic of immature cells to a lysis reagent [9]. However, HPCs can only be utilized in laboratories using the Sysmex analyzer.

Advia 2120i (Siemens Healthcare Diagnostics, Deerfield, IL, USA) differentiates leukocyte subfractions using two channels: enzyme activity determination in the myeloperoxidase channel and nuclear complexity determination in the nuclear lobularity channel. The delta neutrophil index (DNI) is calculated by subtraction of the percentage of polymorphonuclear cells determined in the nuclear lobularity channel from the sum of the percentages of neutrophils and eosinophils, determined in the myeloperoxidase channel [10]. Therefore, the DNI reflects the immature leukocyte subfraction, which has myeloperoxidase activity and lacks nuclear lobularity [11].

Unicel DxH800 (Beckman Coulter, Miami, FL, USA) uses electrical impedance, radiofrequency conductivity, and light scattering to count and differentiate leukocyte subpopulations. Through the measurement data referred to above, 42 cell morphology parameters, which reflect the cell size and internal physical and chemical compositions, are acquired [6]. An increase of the immature leukocyte subfraction in a sample might lead to an increase in variation of the volume and conductivity of each leukocyte subpopulation.

These parameters from the Siemens and Coulter hematology analyzers have been investigated for their diagnostic and prognostic impact on diseases or states characterized by changes in leukocyte morphology, such as infection, sepsis, hematologic disorders, and granulocyte-colony stimulating factor (G-CSF) administration [11–15]. In this study, we aimed to investigate whether cell morphology parameters from the Siemens and Coulter analyzers can also be used for the prediction of the CD34+ cell count after mobilization.

## Materials and methods

### Patients and stem cell mobilization

From July 2013 to January 2016, 53 healthy donors for allogeneic HSC transplantation and 72 patients with hematologic malignancy undergoing autologous transplantation were prospectively recruited for this study. This study was approved by the institutional review board of the Severance Hospital, Yonsei University College of Medicine, Seoul, Korea. Written informed consent was obtained from all participants in accordance with the Declaration of Helsinki. For all healthy donors and 15/72 patients, the standard dose of G-CSF (10 μg/kg/day) was used for mobilization and collection was initiated on the fifth day of G-CSF initiation. For the remaining 57 patients, chemotherapy followed by G-CSF was used for mobilization, and collection was started when the PB white blood cell count increased to 3,000/μL after reaching the nadir. A whole blood sample was taken from all participants immediately prior to the first apheresis. Aliquots of the sample were tested for the CD34+ cell count using flow cytometry, and for the complete blood count using two automated hematology analyzers in parallel: the Unicel DxH800 (Beckman Coulter) and Advia 2120i (Siemens Healthcare Diagnostics). In some of the patients (101/125 patients, 80.8%), CD34+ cell count in apheresis product was acquired by reviewing medical records, and two parameters of HSC yield (CD34+ cells/μL in a product and total CD34+ cells/kg donor body weight) were compared with PB CD34+ cell count.

### Complete blood count

Using DxH800, 42 parameters related to the morphology of leukocytes were obtained from calculations of the mean and standard deviation cell volume, conductivity, and five-angle light scattering measurements [13,14]. Using Advia 2120i, the DNI percentage was obtained as the following formula: DNI (%) = (the leukocyte subfraction assayed in the myeloperoxidase channel by cytochemical reaction)–(the leukocyte subfraction counted in the nuclear lobularity channel by the reflected light beam) [10].

### CD34-positive cell count

The CD34+ cells were enumerated using Stem-Kit reagent (Beckman coulter) according to the manufacturer’s instructions. After vortexing of EDTA specimen, 100 μL aliquot was incubated for 20 min with 20 μL mixture of CD45-FITC monoclonal antibody and CD34-PE monoclonal antibody in the first tube, and with 20 μL mixture of CD45-FITC and IsoClonic control-PE reagent in the second tube. In each tube, 20 μL of viability dye 7-AAD was added to distinguish between viable and nonviable cells. In order to lyse erythrocytes, 2 mL of NH_4_Cl lysing buffer was added and incubated for 10 min. For absolute count of CD34+ cells, 100 μL of stem cell fluorosphere with known concentration was added and incubated for 5 min. After gentle mixing, the product was analyzed on FC500 flow cytometer (Beckman coulter) following manufacturers’ recommendations, based on a gating protocol developed by the International Society of Hematotherapy and Graft Engineering (ISHAGE) [16]. A total of 75,000 CD45+ events were acquired for each sample. Among viable CD45+ and CD34+ events, cells with dim CD45 expression and low sideward light scatter were selected, and events falling outside the cluster of cells with low-to-intermediate forward light scatter were excluded. Absolute CD34+ cell count was calculated using the ratio of fluorosphere to CD34+ cell numbers.

### Statistical analysis

Correlations between continuous variables were analyzed using Pearson’s correlation test after natural-log transformation of variables with skewed distribution. The diagnostic accuracy thresholds of the area under the receiver operating characteristic curve (AUC of ROC) were as follows: 1–0.9, excellent; 0.9–0.8, good; 0.8–0.7, fair; 0.7–0.6, poor; and 0.6–0.5, fail [17]. All P-values were two-sided, and values < 0.05 were considered significant. Statistical analysis was performed using SPSS Statistics version 23.0.0 (IBM Corp., Armonk, NY, USA).

## Results

**Fig 1:**
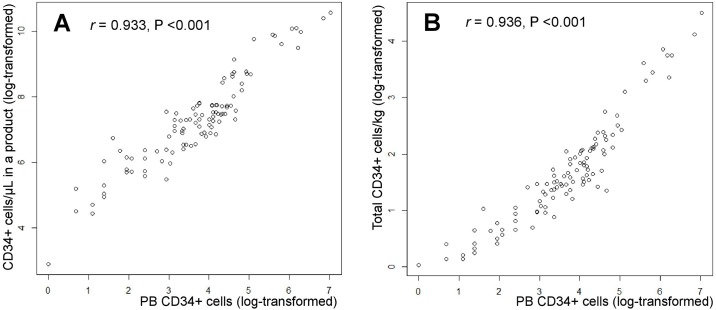
Scatter plots and Pearson’s correlation analyses between (A) PB CD34+ cells/μL and CD34+ cells/μL in a product, and (B) PB CD34+ cells/μL and total CD34+ cells/kg donor body weight (×10^6^). PB, peripheral blood.

This study included a total of 125 subjects, including 53 healthy donors and 72 patients with hematologic malignancy. On the first apheresis day, the median number of CD34+ cells/μL in the PB was 46 (interquartile range, 26–84). The PB CD34+ cell count before apheresis was highly correlated with CD34+ cells/μL in a product (*r* = 0.933, P < 0.001) and total CD34+ cells/kg donor body weight (*r* = 0.936, P < 0.001) (Fig 1).

Among a total of 43 cell morphology parameters, nine parameters: the DNI from Advia 2120i, the standard deviation of volume of neutrophils (SD-V-NE), standard deviation of volume of monocytes (SD-V-MO), standard deviation of conductivity of neutrophils (SD-C-NE), standard deviation of conductivity of monocytes (SD-C-MO), mean conductivity of neutrophils (MN-C-NE), mean conductivity of monocytes (MN-C-MO), standard deviation of axial light loss of neutrophils (SD-AL2-NE), and standard deviation of axial light loss of monocytes (SD-AL2-MO) from DxH800 showed significant correlations with the PB CD34+ cell count after mobilization (Table 1). Next, we calculated the AUCs of the six cell morphology parameters for prediction of each target point of the PB CD34+ cell count. The ROC curve analysis revealed that SD-V-NE, SD-C-NE, and SD-C-MO showed good or fair AUCs in the prediction of several different CD34+ cell count targets.

**Table 1:** Receiver operating characteristics analysis using cell morphology parameters for prediction of the CD34+ cell count.

			AUC for target CD34+ cells/μL in PB			
Parameters^a^	*r*^b^	P-value	< 15, ≥ 15 (n=20, 105)	< 20, ≥ 20 (n=25, 100)	< 25, ≥ 25 (n=31, 94)	< 30, ≥ 30 (n=39, 86)
DNI	0.416	< 0.001	0.598	0.587	0.617	0.633
SD-V-NE	0.313	< 0.001	0.697	0.716	0.728	0.695
SD-V-MO	0.289	0.001	0.691	0.624	0.631	0.573
SD-C-NE	0.285	0.001	0.845	0.778	0.769	0.704
MN-C-NE	-0.275	0.002	0.588	0.563	0.553	0.540
SD-C-MO	0.256	0.004	0.761	0.699	0.705	0.648
SD-AL2-MO	0.246	0.006	0.606	0.574	0.603	0.573
SD-AL2-NE	0.226	0.01	0.535	0.507	0.530	0.506
MN-C-MO	-0.197	0.03	0.625	0.606	0.567	0.515

AUC, area under the receiver operating characteristics curve; PB, peripheral blood; DNI, delta neutrophil index; SD, standard deviation; V, volume; NE, neutrophil; MO, monocyte; C, conductivity; MN, mean; AL2, axial light loss.

^a^ The remaining 34 parameters with a P-value of Pearson’s correlation test ≥ 0.05 were omitted.

^b^ Pearson's correlation coefficient between parameters from hematologic analyzers and peripheral blood CD34+ cell count.

## Discussion

In this study, we identified cell morphology parameters for predicting the PB CD34+ cell count after mobilization. These parameters can be acquired during a complete blood count test, and there is no need for additional samples, reagents, and analysis time. Rapid prediction of mobilization failure enables poor mobilizer to use alternative strategies, such as immediate salvage plerixafor [18].

In our dataset, PB CD34+ cell count before apheresis was highly correlated with HSC yields. Therefore, we used PB CD34+ cell count as a reference value for deciding apheresis initiation.

DNI, SD-V-NE, SD-V-MO, SD-C-NE, SD-C-MO, MN-C-NE, MN-C-MO, SD-AL2-NE, and SD-AL2-MO showed positive or negative correlations with the PB CD34+ cell count in the present study. Lee *et al* reported that SD-V-NE, SD-V-MO, SD-C-NE, SD-C-MO, MN-C-NE, MN-C-MO, SD-AL2-NE, and SD-AL2-MO were significantly increased in a G-CSF administered group, as compared with in the control group [13]. Golubeva *et al* reported that MN-V-NE and SD-V-NE correlated with the CD34+ cell count [5]; however, only SD-V-NE significantly correlated with the CD34+ cell count in our study. These markers can be regarded as reflecting a left shift of leukocytes, rather than a direct measurement of stem cells in a sample. On the other hand, the Sysmex hematology analyzer uses a special lysis reagent for differentiation of immature cells [9]. Therefore, it is possible that HPCs reflect the immature cell population more specifically than parameters from the Siemens and Coulter analyzers. Additional studies are needed to validate these parameters by comparison with HPCs from the Sysmex analyzer in parallel.

Here, we provided evidence that clinical laboratories using hematology analyzers from manufactures other than Sysmex can also use cell morphology parameters to predict the mobilization outcome in clinical practice. In conclusion, SD-V-NE, SD-C-NE, and SD-C-MO can be used as useful indicators for the prediction of optimal timing of HSC collection after mobilization.

## Supporting information

S1 File Table A. Clinical characteristics and laboratory data of the 125 participants in this study. (XLSX)
